# Tuina Analgesia Is Associated With the Modulation of the NCOA4‐Mediated Ferroautophagy–Ferroptosis Pathway in SNL‐Induced Neuropathic Pain Rats

**DOI:** 10.1155/prm/6673176

**Published:** 2026-04-28

**Authors:** Kailong Wang, Peipei Yang, Xiaofeng Gan, Hongliang Tang, Liangyuan Tan, Yueqiang Hu

**Affiliations:** ^1^ Department of Rehabilitation Medicine, The First Affiliated Hospital of Guangxi University of Traditional Chinese Medicine, Nanning, China, gxmu.edu.cn; ^2^ Guangxi Key Laboratory of Molecular Biology of Preventive Medicine of Traditional Chinese Medicine, The First Affiliated Hospital of Guangxi University of Traditional Chinese Medicine, Nanning, China, gxmu.edu.cn; ^3^ Guangxi University of Chinese Medicine, Nanning, China, gxtcm.com; ^4^ Department of Tuina, Fangchenggang Hospital of Traditional Chinese Medicine, Fangchenggang, China

**Keywords:** ferroautophagy, ferroptosis, NCOA4, neuropathic pain, Tuina

## Abstract

**Background:**

Neuropathic pain (NP) has become a major global public health issue. Tuina, a traditional therapy, has been shown to alleviate pain effectively by promoting nerve repair and reducing neuroinflammation. This study aimed to investigate whether Tuina can suppress the ferroptosis‐autophagy pathway to improve NP induced by spinal nerve ligation (SNL) in rats.

**Methods:**

A total of 42 Sprague‐Dawley rats were randomly allocated into seven experimental groups, with six rats in each group: Sham, SNL model, SNL + Sham Tuina, SNL + Tuina (acupoints GB30/GB34/GB39, 5 N pressure, 120 times/min, 18 min/day for 14 days), SNL + si‐NC (empty vector), SNL + si‐NCOA4 (NCOA4 silencing), and SNL + ferrostatin‐1 (10 mg/kg for 7 days). Pain behaviors were assessed using paw withdrawal threshold (PWT) and paw withdrawal latency (PWL). Protein expressions of NCOA4, ferritin heavy chain 1 (FTH1), microtubule‐associated protein 1 light chain 3 (LC3), glutathione peroxidase 4 (GPX4), and acyl‐CoA synthetase long‐chain family member 4 (ACSL4) were detected by the western blot. Immunofluorescence visualized NCOA4‐FTH1 colocalization. The mitochondrial ultrastructure was analyzed by TEM. Fe^2+^ and MDA levels were measured colorimetrically.

**Results:**

Compared with the SNL + Sham group, the Tuina group exhibited a significantly increased PWT (*p* < 0.001) and PWL (*p* < 0.001). IHC analysis revealed that the Tuina intervention reduced the proportion of NCOA4‐positive cells (*p* < 0.001), increased GPX4 expression (*p* < 0.001), and decreased ACSL4 expression (*p* < 0.001) in the spinal dorsal horn. Western blot further confirmed that Tuina downregulated NCOA4, LC3, and ACSL4 protein levels (all *p* < 0.001), while upregulating FTH1 and GPX4 (both *p* < 0.001). IF demonstrated enhanced colocalization of NCOA4 and FTH1 in the Tuina group (*p* < 0.001). TEM observations indicated that Tuina ameliorated ferroptosis‐associated mitochondrial damage, including cristae disruption and membrane rupture. Biochemical assays showed that Tuina significantly reduced the spinal cord Fe^2+^ content (*p* < 0.001) and MDA levels (*p* < 0.001).

**Conclusion:**

Tuina alleviates NP in SNL rats, an effect correlated with the inhibition of NCOA4‐mediated ferritinophagy, decreased iron overload and lipid peroxidation, and attenuated ferroptosis in spinal dorsal horn neurons.

## 1. Introduction

Pain represents a major global public health challenge, characterized by its inherent complexity encompassing both sensory and emotional dimensions [1]. Neuropathic pain (NP), a distinct pain state, manifests primarily through a set of core symptoms: spontaneous discomfort, hyperalgesia (an exaggerated response to painful stimuli), and allodynia (a painful sensation elicited by typically nonpainful stimuli) [2]. This type of pain typically arises from injury to the somatosensory system. NP is a considerable health burden, with epidemiological studies indicating that it affects approximately 3%–17% of people worldwide [3], and conventional analgesics have limited efficacy and are difficult to meet clinical treatment needs [4]. Earlier multiomics investigations have pinpointed transcription factors like AP‐1 as key regulatory elements in the development of hyperalgesia induced by inflammation, thereby highlighting the intricate nature of pain mechanisms [5]. A novel type of programmed cell death, ferroptosis, is implicated as a significant modulator in the pathogenesis of NP [6]. Iron ions, essential trace elements for maintaining life activities, can induce ferroptosis by initiating lipid peroxidation through the Fenton reaction when present in excess [7]. Studies have shown that iron overload is the primary trigger for ferroptosis, with lipid peroxidation being its core mechanism [8]. Specifically, in the chronic sciatic nerve compression injury rat model, significant iron overload is observed, and the use of iron chelators can suppress this process, suggesting that regulating iron levels may influence the progression of pain [9]. Additionally, the elevation of the intracellular labile iron pool is mediated through ferritinophagy, a process where the autophagic degradation of ferritin releases stored iron ions [10].

As an essential regulator of ferritinophagy, nuclear receptor coactivator 4 (NCOA4) enhances the autophagic degradation of ferritin, a process that subsequently leads to the liberation of iron ions [11]. After spinal cord injury, macrophages release redox‐active iron via NCOA4‐mediated ferritinophagy, thereby driving ferroptosis and exacerbating secondary damage [12]. Notably, NCOA4‐mediated ferritinophagy has been directly implicated in driving ferroptosis in degenerative diseases such as intervertebral disc degeneration, highlighting its role in tissue damage processes relevant to chronic pain conditions [13]. Furthermore, mechano‐based interventions such as electroacupuncture have been shown to alleviate NP by inhibiting ferroptosis in ganglia [14] and to protect against neuronal ferroptosis in cerebral ischemia by suppressing ferritinophagy and lysosomal iron release [15]. Therefore, targeting the regulation of the NCOA4‐mediated ferritinophagy‐ferroptosis pathway may provide a new strategy for treating neurodegenerative diseases and potentially NP. As a traditional nonpharmacological therapy, manual therapy has been clinically validated for its analgesic effects [16], particularly in NP caused by nerve injury, where manual therapy effectively alleviates pain by improving nerve repair and reducing neuroinflammation [17, 18].

Based on the above, this study hypothesizes that Tuina may exert its analgesic effects, at least in part, through the modulation of the NCOA4‐mediated ferritinophagy‐ferroptosis pathway, thereby potentially mitigating iron overload and associated neuronal damage. This research aims to investigate this hypothesis and explore the analgesic mechanisms of Tuina in an NP model, with the goal of establishing a foundation for its wider application.

## 2. Materials and Methods

### 2.1. Experimental Animals

This study utilized healthy adult male Sprague‐Dawley (SD) rats with body weights between 250 and 300 g. The animals were sourced from Changsha Tianqin Biotechnology Co., Ltd. (Production License No: SCXK2022‐0011) and subsequently acclimatized in the specific pathogen‐free facility of the Laboratory Animal Center at Guangxi University of Chinese Medicine (License No: SYXK Gui 024‐0004), an environment designed to be rigorously controlled and uncontaminated. For the duration of the study, including the 7‐day acclimatization phase, the animal room conditions were regulated to maintain a stable environment, with a temperature of 22 ± 2°C and humidity at 50 ± 10%. Additionally, a 12‐h light/dark cycle was employed to uphold regular circadian rhythms during the experiment. All animals had free access to water and standard laboratory feed. Furthermore, animal welfare was prioritized through daily replacement of cages and bedding materials to ensure optimal hygiene and health. Ethical approval for this study was obtained from the Animal Research Ethics Committee of Guangxi University of Chinese Medicine (Approval No: DW20240319‐057). The research was conducted in strict accordance with the national “Guidelines for the Management and Use of Experimental Animals (8th Edition, 2011)” and the international ARRIVE Guidelines 2.0.

### 2.2. Spinal Nerve Ligation (SNL)

Rat Model: As outlined in prior studies [19], the rats were anesthetized with isoflurane (Sigma‐Aldrich, I4849), administered via continuous inhalation at an initial flow rate of 2 L/min for induction, which was then reduced to 1 L/min for maintenance. Once anesthetized, the rats were positioned in a prone orientation on the surgical table. Following disinfection with alcohol, a longitudinal incision of approximately 2 cm was made on the left side of the L4‐S1 spinous process. The surgical approach involved a sequential incision through the superficial skin and subcutaneous tissues, followed by the meticulous blunt separation of the underlying left paraspinal muscles and fascia to achieve full exposure of the L6 transverse process. Bone forceps were used to cut the L6 transverse process, allowing for full exposure of the L5 spinal nerve using bone forceps. The L5 spinal nerve was carefully ligated in two layers using noninvasive nylon thread, ensuring that no excessive tension was applied during the procedure. After cleaning and hemostasis, the wound was closed in layers and treated with erythromycin ointment to prevent infection. Postoperatively, rats were kept warm and provided sufficient food and water. In a resting state, rats showed frequent licking and hindlimb hanging on the surgical side, and in an active state, protective hindlimb elevation was observed. No paralysis or dragging of the unaffected limb was noted, and the successful model establishment was confirmed through behavioral testing.

### 2.3. Animal Experimental Grouping

The sample size for this study was estimated using G∗Power 3.1 software (*F* test, one‐way ANOVA). Based on the effect size (Cohen’s *f* = 0.7) obtained from the preliminary experiments, with *α* = 0.05 and statistical power = 0.80, the calculation indicated that at least five samples per group were required. To improve reliability and account for potential sample loss, the final sample size was set to six per group, resulting in a total of 42 samples. All rats weighed 250–300 g at the beginning of the experiment. Body weight was recorded prior to randomization, and animals were allocated using a random number table while ensuring that the mean body weight was balanced across groups. No significant difference in the baseline body weight was observed among the groups. A total of 42 SD rats were randomly allocated to seven distinct experimental groups using a random number table. Each group consisted of six animals and was designated as follows: Sham group: exposed the left L5 spinal nerve without ligation or intervention. SNL group: ligation of the left L5 spinal nerve to establish the SNL group. SNL + Sham Tuina group: fixed SNL rats and performed 18 min of Sham manipulation on both hindlimbs daily for 14 days. Bilateral manipulation was applied to maintain consistency with the clinical practice of Tuina therapy, where both limbs are commonly treated to balance neuromuscular tension and improve overall circulation. In addition, bilateral handling ensured that the animals received comparable restraint and tactile stimulation on both sides, thereby minimizing potential behavioral bias caused by unilateral manipulation. During sham manipulation, the operator maintained a light physical contact with the hindlimb skin using the fingertips, but stimulation was deliberately applied to areas adjacent to, rather than directly over, the acupoints GB30 (Huantiao), GB34 (Yanglingquan), and GB39 (Xuanzhong). No standardized Tuina techniques (such as point pressing, plucking, or kneading) were performed. The applied force was minimal (< 1 N), insufficient to produce therapeutic mechanical stimulation, and no rhythmic pressure or fixed frequency was applied. This procedure was designed to control for nonspecific effects related to animal handling, restraint, and skin contact while avoiding specific acupoint stimulation or mechanical loading. SNL + Tuina group: fixed SNL rats, and manual therapy was applied to the acupoints GB30, GB34, and GB39 using a Pressure Profile System (FingerTPS, USA) for 18 min daily, with 5 N of pressure and a frequency of 120 times/min for each technique (point method, plucking method, and kneading method) for 14 days [19]. Consistent with the sham procedure, Tuina manipulation was performed bilaterally on the hindlimbs to standardize handling conditions across groups. All manipulations were performed by the same trained operator according to a standardized protocol to minimize experimental variability. Stimulation of GB30, GB34, and GB39 along the Gallbladder Meridian has been clinically proven to provide analgesic effects [20]. SNL + si‐NC group: intrathecal injection of the AAV empty vector (si‐NC) at a dose of 1 × 10^12^ v.g, administered once. SNL + si‐NCOA4 group: intrathecal injection of AAV (si‐NCOA4) at a dose of 1 × 10^12^ v.g, administered once. SNL + ferrostatin‐1 group: intraperitoneal injection of ferrostatin‐1 (Merck, Germany, SML0583) (10 mg/kg) for 7 consecutive days [21]. Detection of adenovirus silencing efficiency: The SD rats (*n* = 25) were randomly allocated into five distinct groups, with five rats in each group: sham group, SNL + empty vector group (SNL + si‐NC group), SNL + si‐NCOA4 (130502‐1) group, SNL + si‐NCOA4 (130503‐1) group, and SNL + sh‐NCOA4 (130504‐1) group. Except for the sham operation group, the other groups received intrathecal injections of AAVPHP.eB‐NCOA4 with a viral titer of 1.0 × 10^13^ v.g/mL and a viral dose of 1.0 × 10^12^ v.g. Tissue samples were collected three weeks later for quantitative Real–Time PCR (qPCR) analysis to assess the silencing efficiency. Since AAV requires 2–3 weeks postinjection to achieve stable transgene expression, the animal model was established at 3 weeks after viral injection.

### 2.4. Paw Withdrawal Threshold (PWT) Test

In accordance with previously described and validated methodology [19], mechanical pain sensitivity, as assessed by the PWT, was evaluated at multiple time points: preoperatively (baseline) as well as on postoperative Days 3, 7, 10, and 14. A mechanical paw pressure meter (Nanjing Calvin Bio‐Tech Co., Ltd., China, KW‐CT‐1) was used for this test. Prior to assessment, the rats underwent a 30‐min acclimation period inside transparent plastic chambers. Subsequently, the central region of the left hind paw plantar surface was stimulated vertically through the metal grid floor using an electronic von Frey filament. Positive behavior such as paw licking or withdrawal was used as the endpoint for the stimulus. Measurements were repeated three times at five‐minute intervals, and the average was calculated as the PWT. The behavioral assessments were conducted by experimenters who were blinded to the group allocation of the animals.

### 2.5. Paw Withdrawal Latency (PWL) Test

As described in prior experiments [19], to monitor the progression of thermal pain sensitivity, a longitudinal assessment of the PWL was conducted. Measurements were taken preoperatively to establish a baseline, followed by repeated evaluations on postoperative Days 3, 7, 10, and 14. The test was performed using an intelligent thermal pain meter (Nanjing Calvin Bio‐Tech Co., Ltd., China, KW‐RB). Prior to testing, the rats were allowed to acclimate for 30 min at room temperature (25°C). After the temperature of the device was set to 50°C, the rat was placed in a closed transparent glass box, and a timer was started. When rapid paw lifting or frequent paw licking was observed, the timing was stopped, and the latency was recorded. Each measurement was repeated three times at five‐minute intervals, and the average was taken as the PWL. The PWL tests were also performed by investigators blinded to the treatment groups. At the conclusion of the behavioral testing, all rats were deeply anesthetized via the intraperitoneal injection of sodium pentobarbital (50 mg/kg). The depth of anesthesia was confirmed by the absence of a withdrawal reflex upon firm toe‐pinch, after which the animals were euthanized by cervical dislocation. The L4‐L6 spinal cord segments were harvested and allocated for molecular and histological analyses, respectively. Upon collection, the samples intended for molecular biology analyses were snap‐frozen in liquid nitrogen and subsequently stored at −80°C until processing. Conversely, specimens designated for histological staining underwent fixation in 4% paraformaldehyde for 24 h at 4°C. The personnel performing molecular and histological analyses were also blinded to the group assignments. The L4–L6 spinal cord segments were selected because primary afferent fibers of the sciatic nerve predominantly project to the lumbar dorsal horn, where their central terminals form synaptic contacts with second‐order sensory neurons and initiate nociceptive signal transmission [22]. In the SNL model, injury to the L5 spinal nerve induces pathological changes not only at the injured segment but also in adjacent lumbar segments through cross‐segmental mechanisms involving intact L4 afferents and dorsal horn neuronal plasticity [23, 24]. Previous studies have shown that the dorsal horn of the L4–L6 spinal segments exhibits prominent neuroinflammatory responses and glial activation following L5 SNL, which are key contributors to central sensitization during NP [25–27]. Therefore, these lumbar segments were selected as the primary anatomical region for mechanistic analysis in the present study. Accordingly, the present study focused on molecular and cellular changes in the spinal dorsal horn to investigate the spinal mechanisms underlying Tuina‐mediated analgesia.

### 2.6. qPCR

The L4–L6 spinal cord segments were collected into prechilled PBS (Beijing Solarbio Technology Co., Ltd., China, P1022) and cryopreserved at −80°C. The isolation of total RNA was then commenced by homogenizing the tissue samples with 1 mL of RNAiso Plus reagent (Bai Rui Doctor Biotechnology Co., Ltd., China, 9109) to achieve complete lysis. For phase separation, 200 µL of chloroform was added per 1 mL of the initial lysate. The resulting mixture was then subjected to centrifugation at 12,000 × g for a duration of 15 min at 4°C to separate the phases. The RNA‐containing aqueous phase was combined with an equal volume of isopropanol. After standing at room temperature for 10 min, the sample was centrifuged (12,000 × g, 10 min, 4°C), resulting in the RNA pelleting at the bottom of the tube. The RNA was then dissolved in 30 µL of DEPC‐treated water. To evaluate its concentration and purity, a spectrophotometer (Shanghai Baoyude Scientific Instrument Co., Ltd., China, Micro Drop) was utilized. Following the protocol for the qPCR kit TB Green Premix Ex TaqII (Tli RNaseH Plus, RR820A), amplification was performed using specific primers, with GAPDH serving as the reference gene (Table 1). The PCR procedure was initiated with a predenaturation step (95°C, 30 s). Amplification was carried out for 40 cycles using a two‐step thermal protocol, with each cycle consisting of a 5‐s denaturation step at 95°C followed by a 34‐s combined annealing and extension step at 60°C. The verification of specific product amplification was accomplished via melt curve analysis using the 2^−ΔΔCt^ method [28], and the relative expression of the NCOA4 gene was quantified, with GAPDH utilized as the reference gene for normalization.

**TABLE 1:** Sequences of primers used for PCR amplification.

Target gene	Primer sequence (5′ to 3′)
R‐GAPDH‐F	AGA​CAG​CCG​CAT​CTT​CTT​GT
R‐GAPDH‐R	TGA​TGG​CAA​CAA​TGT​CCA​CT
NCOA4‐F	CGG​AAC​AGT​GAG​CAG​AAT​GA
NCOA4‐R	ATT​CCA​GGT​GGC​GAC​TAA​TG

### 2.7. Hematoxylin and Eosin (H&E) Staining

The L4–L6 spinal cord tissues were preserved using 4% paraformaldehyde and then subjected to dehydration and clearing treatments. The tissues were infiltrated with and embedded in paraffin after ethanol removal with xylene and were subsequently processed into thin sections once the embedding medium solidified. The tissue sections were subjected to deparaffinization, followed by rehydration in graded ethanol solutions (95%, 85%, and 75%) for 5 min each. Following the initial hydration in distilled water, the sections were stained by immersion in hematoxylin for 3 minutes, which was rapidly followed by a 2‐s differentiation step in 1% acid‐alcohol. Subsequently, 1‐s immersion in lithium carbonate solution was applied to achieve bluing, culminating in a 6‐min counterstain with eosin to complete the H&E staining process. During dehydration, the sections were dried in an oven for 3 h, and then, subsequent to a 10‐h dehydration step employing absolute ethanol and clearing in xylene, the samples were coverslipped with a neutral polymer‐based resin. The stained sections were examined and imaged using a Leica DM2000 LED microscope at 400x magnification.

### 2.8. Immunohistochemistry (IHC)

Rat spinal cord tissue sections (4 µm thick) were first subjected to routine dewaxing and rehydration procedures. The sections were washed three times for 5 min each with 0.01 M phosphate‐buffered saline (PBS, pH 7.4). To quench endogenous peroxidase activity, the sections were treated with 3% hydrogen peroxide for 10 min at room temperature, followed by a PBS rinse. Subsequently, a 1‐h block was performed at room temperature using 5% normal goat serum (Solarbio, China, SA163). Primary antibody incubation was then carried out overnight at 4°C using the following: anti‐NCOA4 (Proteintech, 83394‐4‐RR, 1:200), anti‐glutathione peroxidase 4 (GPX4) (Proteintech, 67763‐1‐Ig, 1:1000), and anti–acyl‐CoA synthetase long‐chain family member 4 (ACSL4) (Proteintech, 22401‐1‐AP, 1:50). Following three 5‐min washes with PBS, the sections were exposed for 1 h at room temperature to HRP‐conjugated secondary antibodies: goat anti‐rabbit (Abcepta, ASP1615, 1:300) and goat anti‐mouse (Abcepta, ASP1613, 1:400). Prior to signal visualization, unbound antibodies were removed via three additional PBS washes, and the DAB chromogenic reaction was performed using a commercial kit (Beijing Solarbio Science & Technology Co., Ltd., China, DA1010). Following reaction termination, sections were washed with PBS and stained with HE for contrast. The sections were mounted using neutral gum (Shanghai Specimen Model Factory, China, S3006) and examined under a microscope.

### 2.9. Western Blot

L4–L6 spinal cord tissue samples were lysed in 300 µL of lysis buffer (Shanghai Biotian Biotechnology Co., Ltd., China, P0013B), supplemented with PMSF, and incubated on ice for 30 min. Grinding beads were added, and the samples were ground twice for 30 s (4°C) and 6 m/s. Centrifugation of the samples was carried out at 12,000 × g for 10 min under refrigeration (4°C). The supernatant obtained was then subjected to protein concentration determination using the bicinchoninic acid (BCA) method, wherein standards and samples were dispensed into a 96‐well plate and incubated with BCA working solution (Shanghai Biotian Biotechnology Co., Ltd., China, P0012) at 37°C (30 min). The incubation was followed by the measurement of the absorbance at 562 nm, from which the protein concentration was derived. Based on the BCA assay results, equal amounts of total protein (30 µg) were loaded for all samples. The protein samples were prepared for electrophoresis by dilution in 5× loading buffer at a 4:1 ratio, followed by heat denaturation at 100°C for 10 min. Protein separation was subsequently performed using SDS–PAGE (Beijing Solarbio Technology Co., Ltd., China, P1200). Protein transfer was performed onto a methanol‐activated PVDF membrane (Merck Millipore, Germany, IPVH00010) after the electrophoresis step. Following transfer, after being blocked, the membrane underwent an overnight incubation with primary antibodies at a temperature of 4°C: targeting NCOA4 (ab314553), ferritin heavy chain 1 (FTH1) (ab183781), light chain 3 (LC3) (ab18709), GPX4 (ab125066), ACSL4 (ab240135) from Abcam, UK, each at a 1:1000 dilution, and GAPDH (10068‐1‐AP) from Wuhan Sanying Biotechnology Co., Ltd., China, which served as an internal loading control for normalization. The following day, HRP‐conjugated secondary antibodies (Beijing Ximeijie Technology Co., Ltd., China 074‐1506) were introduced and incubated for 60 min at room temperature. Following TBST washing (Beijing Solarbio Technology Co., Ltd., T1082), protein bands were visualized using ECL (Shanghai Biotian Biotechnology Co., Ltd., P0018S), and their intensities were analyzed with ImageJ for statistical evaluation.

### 2.10. Immunofluorescence (IF)

Spinal cord tissues from L4–L6 were fixed in 4% paraformaldehyde, dehydrated, cleared, and then embedded in paraffin. Following hardening, tissue sections were prepared and baked at 60°C for 30 min. Deparaffinization was done with xylene and ethanol, followed by hydration with distilled water. Tissue sections were subjected to antigen retrieval through 8 min of microwave heating in citrate buffer (pH 6.0), followed by rinsing with PBS. To eliminate endogenous peroxidase activity, the samples were treated with 3% H_2_O_2_ for 10 min and washed with PBS. To minimize autofluorescence, tissues were incubated with quenching solution A for 30 min at room temperature, followed by a distilled water rinse. The sections were then blocked with serum for 20 min and incubated overnight at 4°C with primary antibodies targeting FTH1 (Proteintech, 10727‐1‐AP, 1:200) and NCOA4 (Proteintech, 83394‐4‐RR, 1:200). The following day, fluorescent secondary antibodies (Servicebio, GB22303 for FTH1 and GB22301 for NCOA4, both at 1:200 dilution) were applied and incubated at 37°C for 1 min and then washed with PBS. For nuclear staining, the sections were treated with DAPI for 5 min under dark conditions at room temperature. After a PBS rinse, the samples were processed with Servicebio’s spontaneous fluorescence quencher solution B (G1222) for 5 min. The slides were rinsed for 3 min under running water, mounted, and examined using an Olympus BX53 fluorescence microscope at 400× magnification.

### 2.11. Transmission Electron Microscope (TEM)

L4–L6 spinal cord tissue samples were collected carefully to avoid mechanical damage, with a size of approximately 1 mm^3^. Samples were immediately placed in electron microscopy fixative, cut into small blocks, and fixed at 4°C. Tissue samples were initially washed three times with 0.1 M phosphate buffer (pH 7.4) and subsequently fixed in 1% osmium tetroxide for 4 h at room temperature under dark conditions, followed by additional buffer washes. A graded ethanol series (30%, 50%, 70%, 80%, 95%, 100%) was then used for dehydration, with 20‐min incubations at each concentration, followed by two 15‐min incubations in pure acetone. For the embedding process, infiltration was conducted sequentially using acetone: 812 embedding medium mixtures before final treatment with pure 812 medium for 6 h. Following placement in embedding molds and infiltration, 812 resin samples underwent stepped polymerization (37°C overnight and then 60°C for 48 h). Ultrathin sectioning to 70 nm was performed using an ultramicrotome (Thermo Fisher Scientific, USA, Leica UC7), with sections transferred to 150‐mesh copper grids. Staining involved sequential treatment with 2% uranyl acetate (8 min) and 2.6% lead citrate (8 min), separated by washing steps. Ultrastructural analysis was conducted using a TEM (Hitachi, Japan, HT7800/HT7700) at 8000x magnification.

### 2.12. Fe^2+^ Content Measurement

L4–L6 spinal cord tissue samples were homogenized in 100 µL lysis buffer and shaken for 20 s, followed by 2 h of shaking at 5°C. The samples underwent centrifugation at 12,000 × g for 5 min, after which the supernatant was collected for further analysis. The iron standard stock solution (3 mM) was serially diluted to various concentrations using the provided reagent kit. The reaction mixture was prepared by combining the buffer and a 4.5% potassium permanganate solution in equal proportions. When the Fe^2+^ concentration in the samples was high, the lysis buffer was diluted. The reaction mixtures were added to each tube, which were then incubated at 60°C for 1 h with tightly closed caps. Following equilibration to room temperature, the samples were centrifuged to consolidate all liquid at the bottom of the tube. The assay procedure commenced with the addition of iron ion detection reagent (Chengdu Boshi Biotechnology Co., Ltd., China, DIFE‐250). The reaction was conducted at room temperature for 30 min following thorough mixing. After centrifugation, 200 µL of the supernatant was placed into a 96‐well plate for spectrophotometric analysis at 590 nm. Quantitative determination of Fe^2+^ concentration (µM) was achieved through interpolation from a standard curve.

### 2.13. Malondialdehyde (MDA) Content Measurement

Quantification of the MDA content was performed using the corresponding assay kit (Beyotime, A003‐1‐1). At the beginning of the experiment, 1 g of L4‐L6 spinal cord tissue samples was homogenized in PBS (1:10, tissue: solution). The homogenized samples were kept on ice, and the centrifuge tubes were sealed with holes for gas release. Samples were centrifuged at 3500 × g for 10 min to collect the supernatant. Following the MDA test kit instructions (Kelu Biotechnology Co., Ltd., Wuhan, China, BC021‐2), samples were combined with the MDA reaction solution. After the reaction, the samples were rapidly cooled with cold water and centrifuged to remove precipitates, and then, the supernatant was collected for further use. Absorbance at 532 nm was recorded with a Thermo Fisher Scientific Multiskan FC microplate reader. The MDA concentration in the samples was calculated using the standard curve method, with the results expressed in nmol/mL.

### 2.14. Data Analysis

The statistical analyses were performed utilizing SPSS 25.0 (IBM, USA) and GraphPad Prism 10 (GraphPad Software, San Diego, CA, USA), with quantitative data expressed as the mean ± standard deviation (mean ± SD). Error bars in all figures represent the SD. Prior to all statistical comparisons, data were tested for normality using the Shapiro–Wilk test and for the homogeneity of variance using Levene’s test. For data conforming to these parametric assumptions, group comparisons were made using a one‐way repeated measures ANOVA. When assessing the effects of both group and time factors, a two‐way repeated measures ANOVA was applied to evaluate main effects and their interactions. In instances where significant differences were detected by ANOVA, post hoc comparisons between specific groups were performed using Tukey’s HSD test for all pairwise comparisons. For data that did not meet the assumptions of normality or homogeneity of variance, the Kruskal–Wallis H test followed by Dunn’s post hoc test was applied. A probability (*p*) value of less than 0.05 was considered to indicate the statistical significance. No animals died or were excluded from the study; therefore, data from all animals that entered the experimental protocol were included in the final analysis.

## 3. Results

### 3.1. NCOA4 Is Highly Expressed in Spinal Cord of SNL‐Induced NP Rats and Is Effectively Downregulated by NCOA4 Silencing, Ferrostatin‐1, or Tuina intervention

Figure 1(a) illustrates the left L5 SNL model. NCOA4 silencing led to a significant reduction in its expression in the spinal cord across all experimental groups (*p* < 0.001). At the transcriptional level, the administration of si‐NCOA4‐130502, si‐NCOA4‐130503, and si‐NCOA4‐130504 each resulted in substantially lower NCOA4 mRNA levels compared to the control group (*p* < 0.001) (Figure 1(b)). This downregulation was further verified at the protein level by IHC, which showed suppressed NCOA4 expression in the SNL + ferrostatin‐1 group relative to the SNL group, as well as in the SNL + Tuina relative to the SNL + Sham Tuina (*p* < 0.001) (Figures 1(c), 1(d)).

FIGURE 1Regulation of NCOA4 expression in the SNL‐induced NP model and the effect of Tuina intervention. (a) SNL model construction and group treatments; (b) expression of NCOA4 mRNA. (c) Immunohistochemistry of NCOA4 (400x). (d) Positive cell rate of NCOA4 (%). *n* = 6 rats/group; data are presented as mean ± SD and analyzed by one‐way ANOVA followed by Tukey’s HSD test. Error bars represent the SD. Scale bar: 50 µm. ^∗∗∗^ *p* < 0.001. SNL: Spinal nerve ligation, NCOA4: nuclear receptor coactivator 4.

**Figure:**
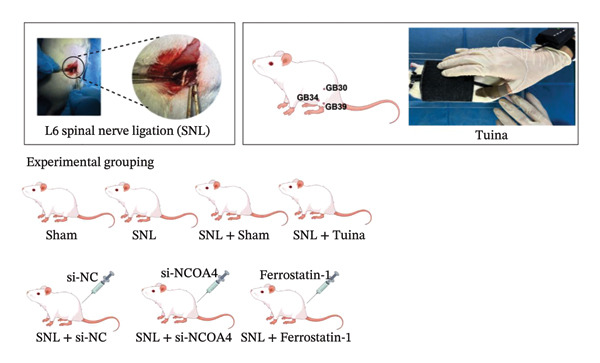
(a)

**Figure:**
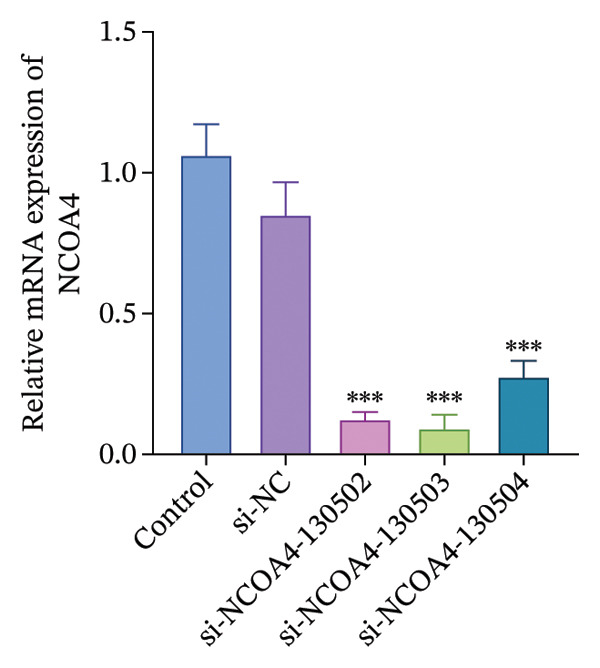
(b)

**Figure:**
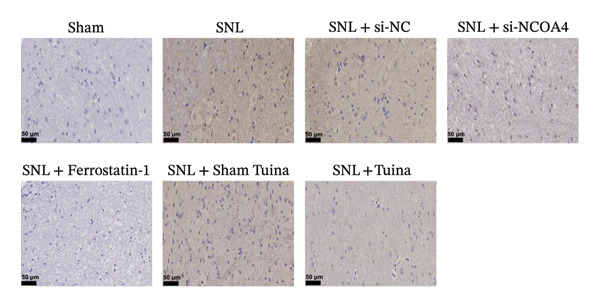
(c)

**Figure:**
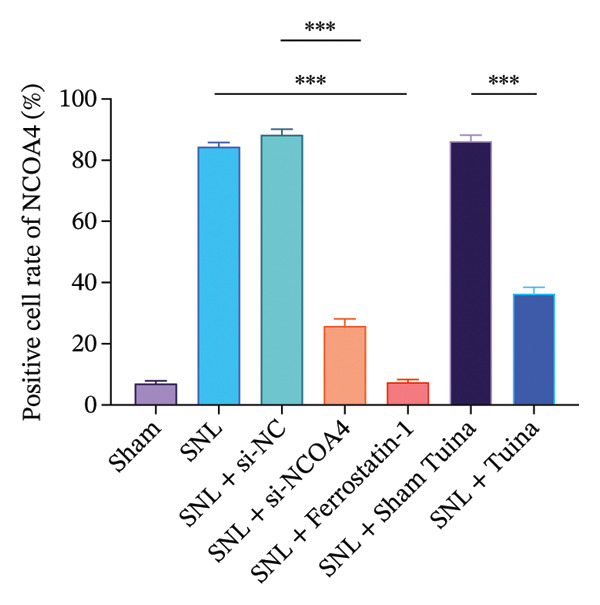
(d)

### 3.2. Ferrostatin‐1, NCOA4 Silencing, and Tuina Improve Mechanical Hyperalgesia and Spinal Cord Injury in SNL Model Rats by Inhibiting Ferroptosis

Experimental findings indicated a significant reduction in the PWT of SNL rats from Day 7 (*p* < 0.01). However, treatments with ferrostatin‐1, NCOA4 silencing, and Tuina significantly increased PWT from Day 10 (*p* < 0.001) (Figure 2(a)). PWL revealed a marked reduction in the SNL group (*p* < 0.01). From Day 10 onwards, a notable increase in PWL was observed after treatment with ferrostatin‐1, NCOA4 silencing, or Tuina (*p* < 0.001). The Tuina and SNL + si‐NCOA4 groups showed a significant increase in PWL (Figure 2(b)). Figures 2(c) and 2(d)) display the representative images captured during the PWT and PWL tests. On histological evaluation, the spinal cord in the SNL group displayed severe tissue injury, manifesting as condensed nuclei, degeneration, and loss of normal tissue architecture. In the SNL + si‐NCOA4 and SNL + ferrostatin‐1 groups, these pathological changes were significantly improved. Furthermore, the Tuina group also showed similar protective effects, with a relatively intact tissue structure and normal cell morphology (Figure 2(e)).

FIGURE 2Effects of different interventions on NP behaviors and spinal cord histomorphology in SNL model rats. (a) PWT in each group; (b) PWL in each group; (c) PWT test photography; (d) PWL test photography; (e) HE staining of spinal cord tissue (400x). *n* = 6 rats/group; data are presented as mean ± SD and analyzed by two‐way repeated measures ANOVA followed by Tukey’s HSD test. Error bars represent the SD. Scale bar: 50 µm. ^∗^: SNL group vs Sham group; ^#^: SNL + Tuina group vs SNL + Sham Tuina group. ^∗^Significance levels are denoted as follows: ^∗^ *p* < 0.05, ^∗∗^ *p* < 0.01, ^∗∗∗^ *p* < 0.001; ^#^ *p* < 0.05, ^##^ *p* < 0.01, ^###^ *p* < 0.001. SNL: Spinal nerve ligation, NCOA4: nuclear receptor coactivator 4, PWT: paw withdrawal threshold, PWL: paw withdrawal latency.

**Figure:**
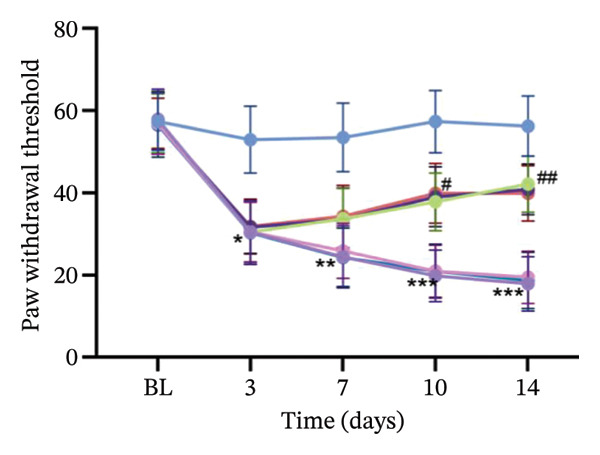
(a)

**Figure:**
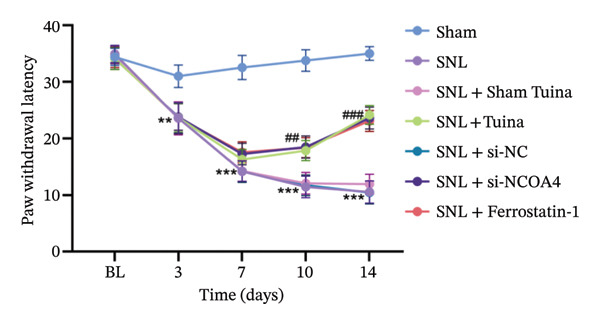
(b)

**Figure:**
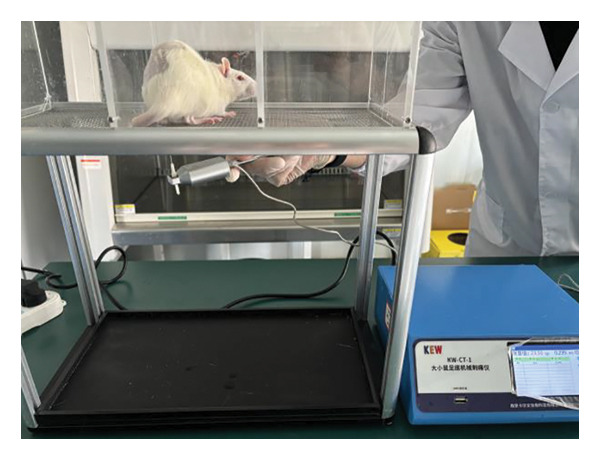
(c)

**Figure:**
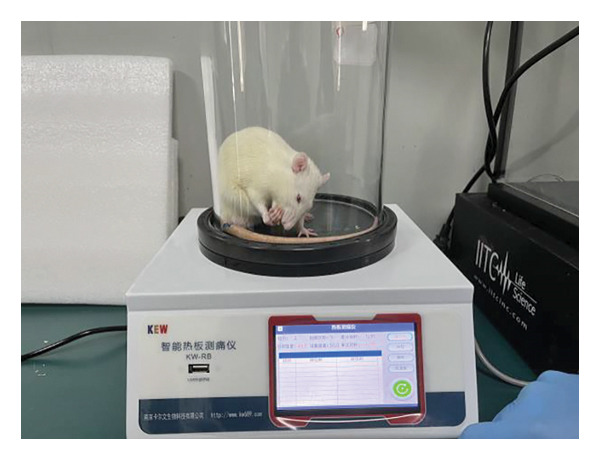
(d)

**Figure:**
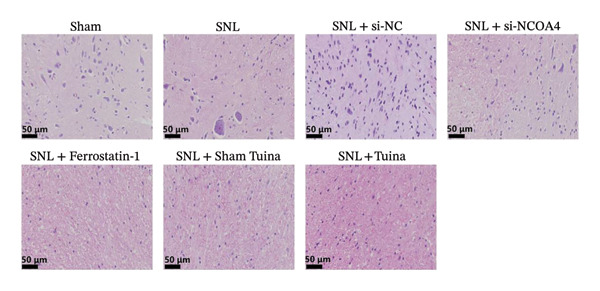
(e)

### 3.3. Tuina Relieve NP by Regulating Ferroptosis‐Related Protein

Western blot analysis revealed significantly elevated expression levels of NCOA4, LC3, GPX4, and ACSL4 proteins in the SNL group compared to the Sham group, with all differences being highly statistically significant (*p* < 0.001) (Figures 3(a), 3(b), 3(c), 3(d), 3(e), and 3(f)). In the SNL + intervention groups (ferrostatin‐1, si‐NCOA4, and Tuina), protein expression was significantly downregulated (*p* < 0.001), with the Tuina and si‐NCOA4 groups, showing marked reductions in NCOA4, FTH1, LC3, and related proteins (*p* < 0.001). Notable differences were identified between the SNL + Sham Tuina and SNL + Tuina (*p* < 0.001) (Figures 3(a), 3(b), 3(c), 3(d), 3(e), and 3(f)).

FIGURE 3Effects of different interventions on the expression of key proteins in the ferroptosis and ferritinophagy pathways in the spinal cord of SNL model rats. (a) Western blot test; (b) NCOA4 expression level; (c) FTH1 expression level; (d) LC3 expression level; (e) GPX4 expression level. (f) ACSL4 expression level. *n* = 3 (biological replicates); data are presented as mean ± SD and analyzed by one‐way ANOVA followed by Tukey’s HSD test. Error bars represent the SD. ^∗∗∗^ *p* < 0.001. NP: Neuropathic pain, SNL: spinal nerve ligation, NCOA4: nuclear receptor coactivator 4, FTH1: ferritin heavy chain 1, LC3: microtubule‐associated protein 1A/1B‐light chain 3, GPX4: glutathione peroxidase 4, ACSL4: acyl‐CoA synthetase long‐chain family member 4.

**Figure:**
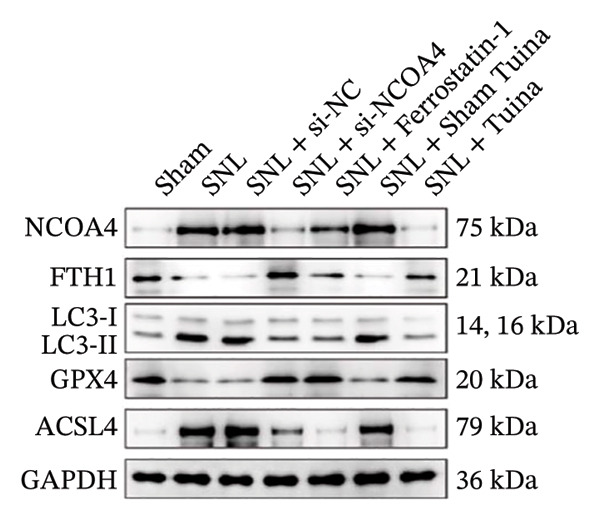
(a)

**Figure:**
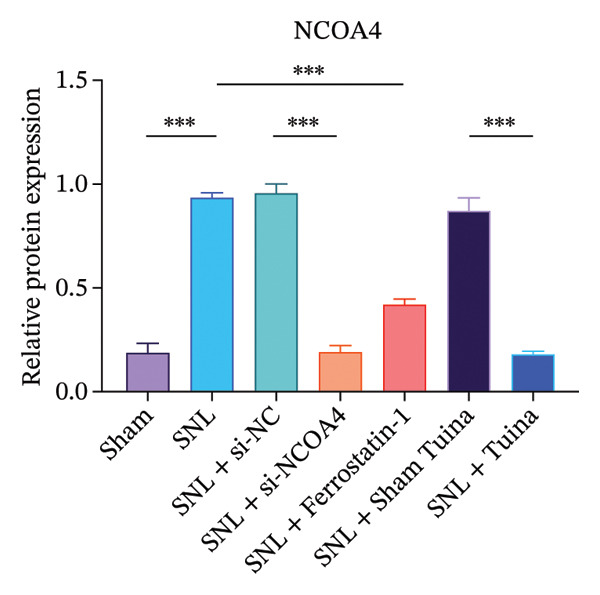
(b)

**Figure:**
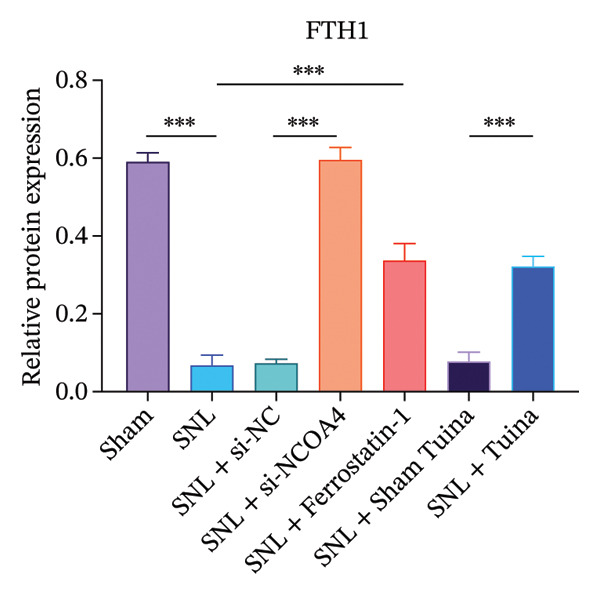
(c)

**Figure:**
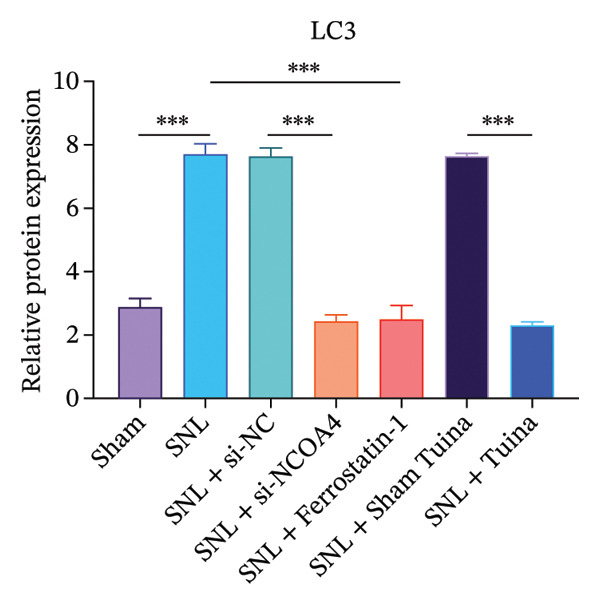
(d)

**Figure:**
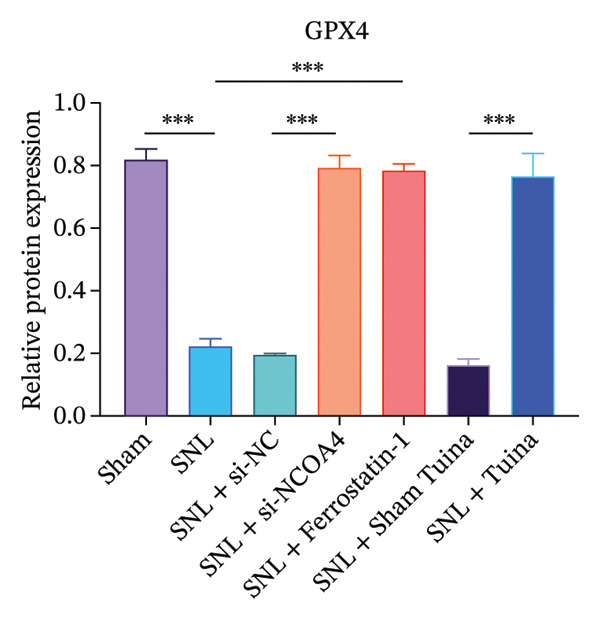
(e)

**Figure:**
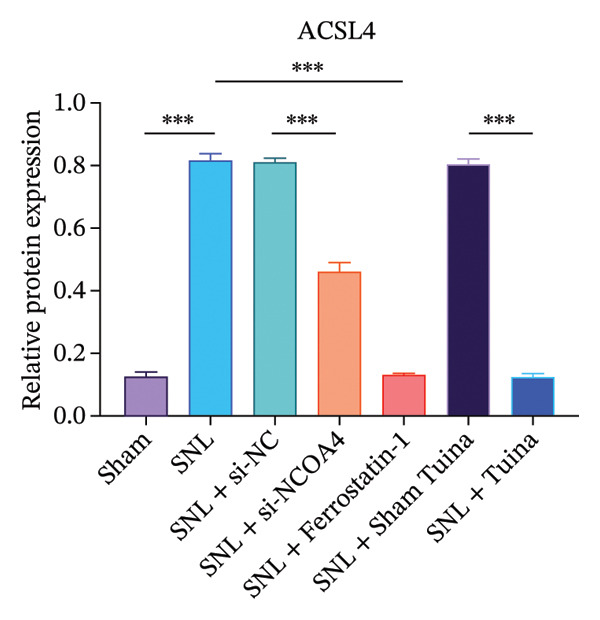
(f)

### 3.4. Expression and Colocalization of NCOA4 and FTH1 in the Dorsal Horn of the Spinal Cord in Rats

IF staining demonstrated a markedly higher percentage of NCOA4‐positive cells in the SNL group relative to the SNL + ferrostatin‐1 group (*p* < 0.001) (Figures 4(a), 4(b)). Conversely, the proportion of FTH1‐positive cells exhibited an inverse pattern, being significantly lower in the SNL group (*p* < 0.001) (Figures 4(a), 4(b)). This reciprocal relationship was corroborated in the SNL + si‐NCOA4 group, which showed a substantial decrease in NCOA4‐positive cells paralleled by a significant increase in FTH1‐positive cells (*p* < 0.001) (Figures 4(b), 4(c)). Furthermore, compared to the SNL + Sham Tuina group, the SNL + Tuina intervention resulted in suppressed NCOA4 expression alongside enhanced FTH1 expression (*p* < 0.001) (Figures 4(b), 4(c)). Colocalization analysis further indicated a significantly stronger interaction between NCOA4 and FTH1 in the SNL + Tuina group than in the SNL + Sham Tuina (*p* < 0.001) (Figure 4(d)).

FIGURE 4Effects of different interventions on the expression and colocalization of ferroautophagy‐related proteins NCOA4 and FTH1 in the spinal dorsal horn of SNL model rats. (a) Immunofluorescence staining; (b) NCOA4 expression level; (c) FTH1 expression level; (d) Colocalization percentage (yellow/blue) (%). *n* = 3 (biological replicates); data are presented as mean ± SD and analyzed by one‐way ANOVA followed by Tukey’s HSD test. Error bars represent the SD. Scale bar: 100 µm ^∗∗∗^ *p* < 0.001. SNL: Spinal nerve ligation, NCOA4: nuclear receptor coactivator 4, FTH1: ferritin heavy chain 1.

**Figure:**
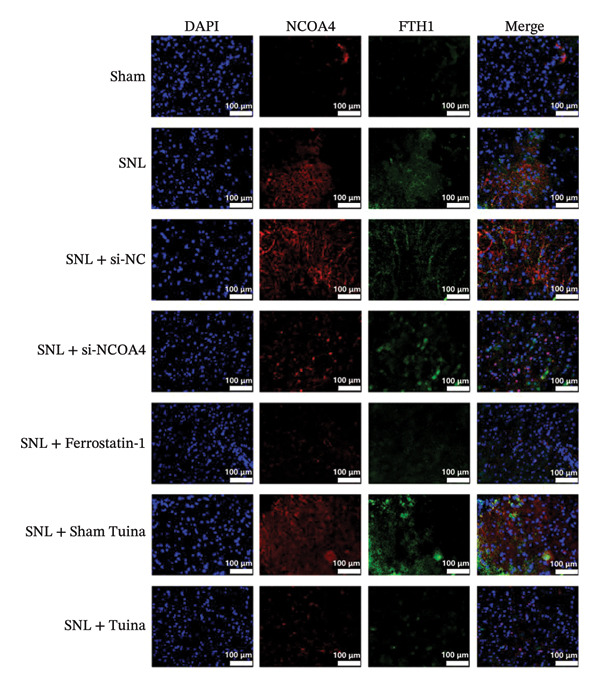
(a)

**Figure:**
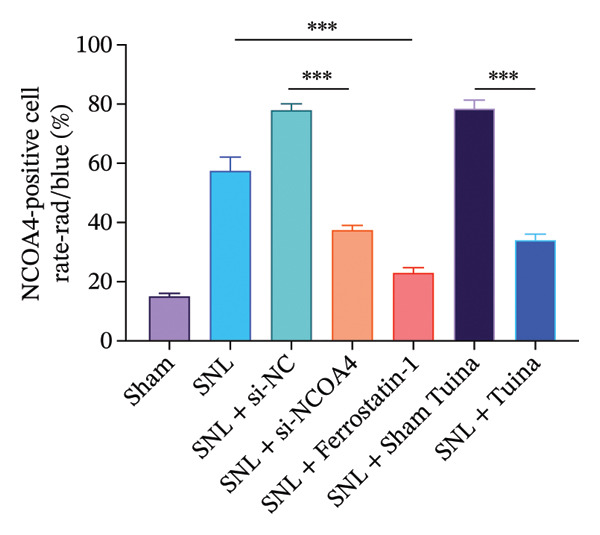
(b)

**Figure:**
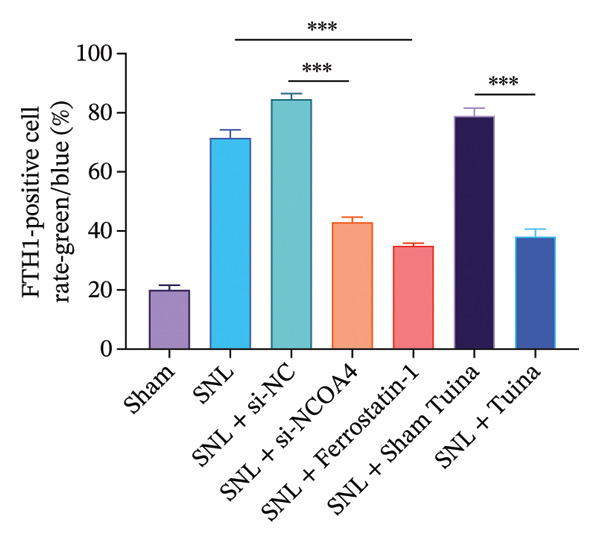
(c)

**Figure:**
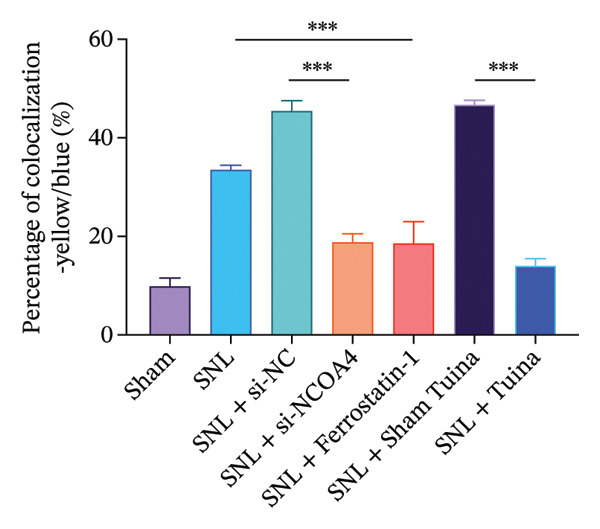
(d)

### 3.5. Tuina Relieve NP by Improving Mitochondrial Damage and Inhibiting Ferroptosis

Experimental findings revealed notable ultrastructural variations in the dorsal horn cells of the rat spinal cord among the groups. The SNL group showed notable mitochondrial damage relative to the Sham group, including swelling, cristae disruption, compromised outer membrane integrity, and a rise in autophagic vacuoles in the cytoplasm, aligning with ferroptosis morphology. Mitochondrial damage in both the SNL + si‐NC and SNL + Sham Tuina groups resembled that of the SNL group, characterized by damaged cristae and vacuolar degeneration. The autophagosome count remained elevated without notable improvement or decline. In the SNL + si‐NCOA4 group, mitochondrial damage was relatively alleviated, with reduced swelling and partial restoration of mitochondrial cristae (Figure 5(a)).

FIGURE 5Effects of different interventions on ferroptosis‐related indicators (Fe^2+^, MDA) and neuronal ultrastructure in the spinal cord of SNL model rats. (a) Ultrastructural changes in dorsal horn cells of the rat spinal cord (8000x); (b) changes in the Fe^2+^ content in the rat spinal cord; (c) Changes in the MDA content in the rat spinal cord. *n* = 3 (biological replicates); data are presented as mean ± SD and analyzed by one‐way ANOVA followed by Tukey’s HSD test. Error bars represent the SD. Scale bar: 1 µm. ^∗∗∗^ *p* < 0.001. SNL: Spinal nerve ligation, MDA: malondialdehyde.

**Figure:**
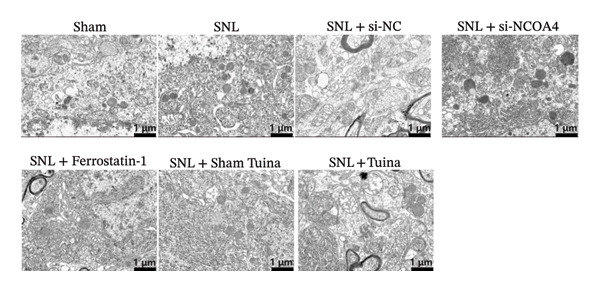
(a)

**Figure:**
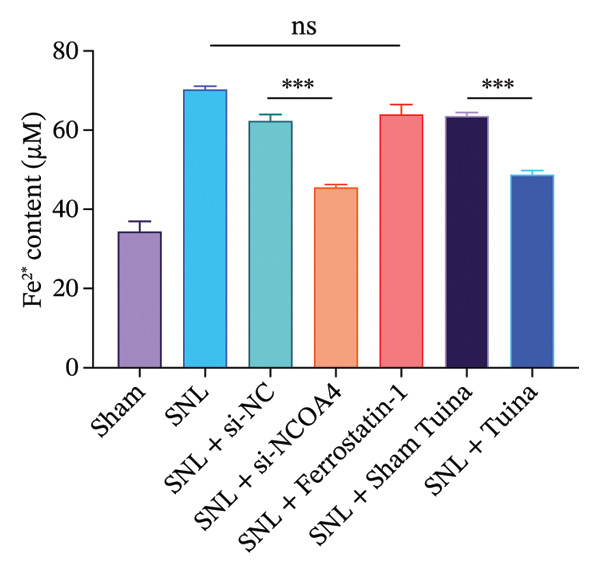
(b)

**Figure:**
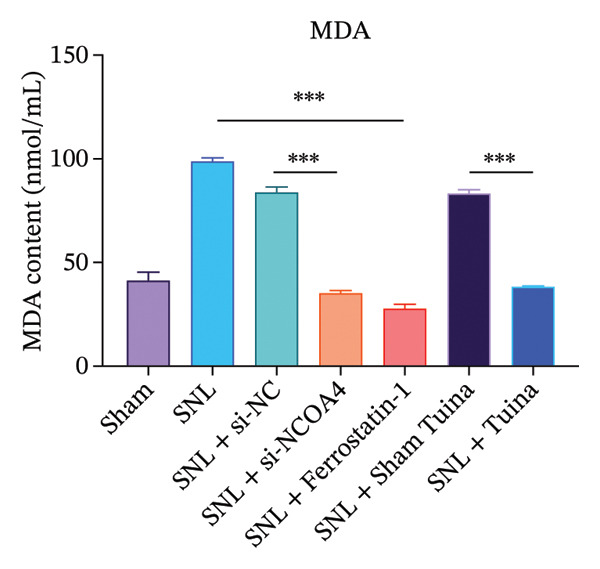
(c)

Quantitative analysis revealed a substantial rise in the Fe^2+^ content within the SNL group compared to the Sham controls (*p* < 0.001), This increase was, however, markedly attenuated in both the SNL + si‐NCOA4 and SNL + Tuina groups (*p* < 0.001). Further analysis revealed that Fe^2+^ levels differed significantly between the SNL + Tuina and SNL + Sham Tuina (*p* < 0.001) (Figure 5(b)). Similarly, the MDA level was significantly elevated in the SNL group but was suppressed in the SNL + si‐NCOA4, SNL + ferrostatin‐1, and SNL + Tuina groups (*p* < 0.001). The effectiveness of the Tuina intervention was further supported by a significant reduction in MDA relative to the Sham Tuina control (*p* < 0.001) (Figure 5(c)).

**FIGURE 7:**
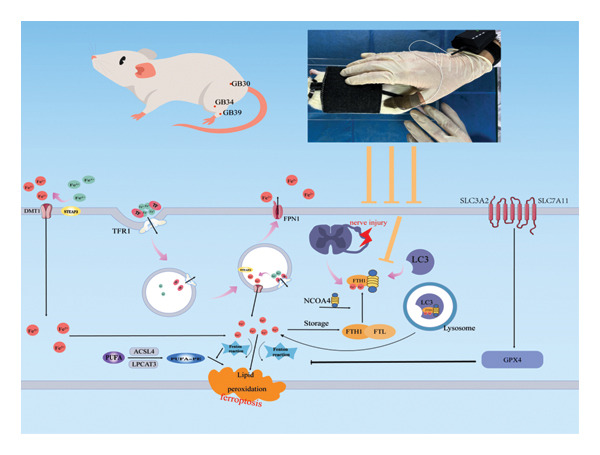
Research mechanism diagram.

IHC staining indicated a marked upregulation of GPX4 in the SNL + ferrostatin‐1 group relative to the SNL group (*p* < 0.001). Likewise, GPX4 levels were substantially higher in the SNL + Tuina group than in the SNL + Sham Tuina group (Figures 6(a) and 6(b)). Conversely, a significant downregulation of ACSL4 was observed in the SNL + ferrostatin‐1 group compared to the SNL group (*p* < 0.001). Consistent with this trend, a significant reduction in ACSL4 expression was observed in the SNL + Tuina group compared to the SNL + Sham Tuina (*p* < 0.001) (Figures 6(c) and 6(d)). Figure 7 shows the mechanism diagram of this study.

FIGURE 6Effects of different interventions on the expression of GPX4 and ACSL4 in the spinal dorsal horn of SNL model rats. (a) Immunohistochemistry of GPX4 (400x); (b) positive cell rate of GPX4 (%). (c) Immunohistochemistry of ACSL4 (400x). (d) Positive cell rate of ACSL4 (%). *n* = 3 (biological replicates); data are presented as mean ± SD and analyzed by one‐way ANOVA followed by Tukey’s HSD test. Error bars represent the SD. Scale bar: 50 µm. ^∗∗∗^ *p* < 0.001. SNL: Spinal nerve ligation, GPX4: glutathione peroxidase 4, ACSL4: acyl‐CoA synthetase long‐chain family member 4.

**Figure:**
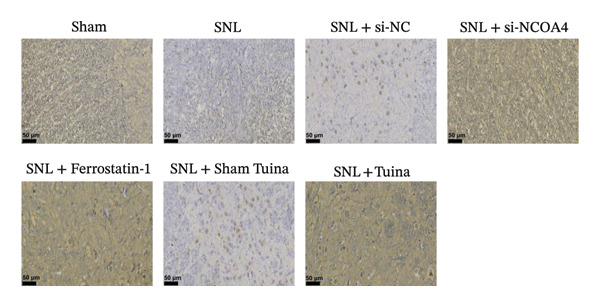
(a)

**Figure:**
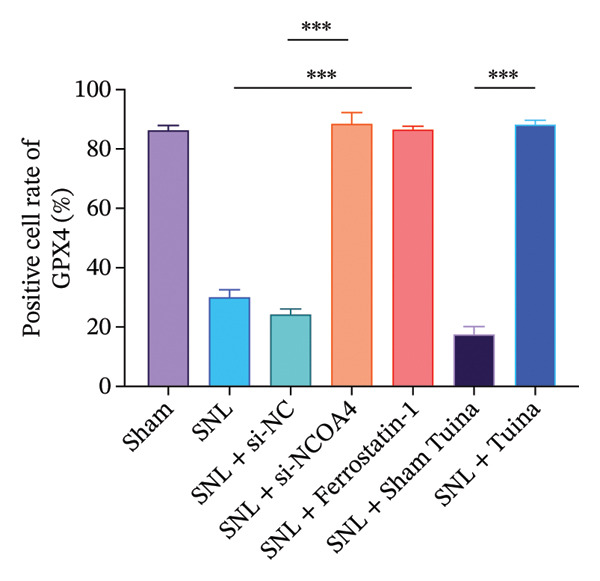
(b)

**Figure:**
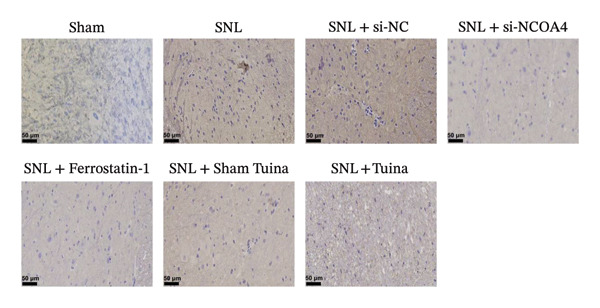
(c)

**Figure:**
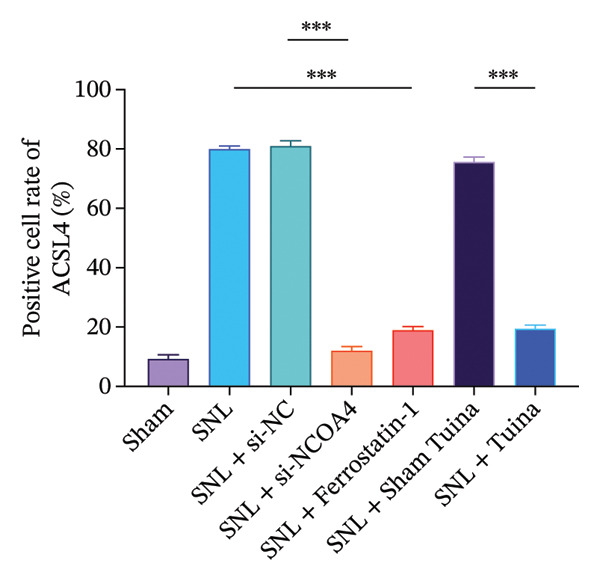
(d)

## 4. Discussion

Research shows that ferroptosis is a novel strategy for regulating NP [6]. During ferroptosis, intracellular iron overload is a key initiating factor, and lipid peroxidation triggered by this overload is the direct driving force [8]. The continuous buildup of Fe^2+^ fuels the Fenton reaction, which serves as a major source of hydroxyl radicals and other reactive oxygen species. This oxidative stress triggers lipid peroxidation, leading to increased lipid peroxide levels [29–31], The resulting damage to cellular membrane integrity causes cell death, a process linked to neurological disorders [32]. In rats with chronic sciatic nerve compression injury and brachial plexus avulsion‐induced NP, iron metabolism disorders promoted intracellular iron overload, leading to time‐dependent mechanical and thermal hypersensitivity [9, 33]. Previous research indicates that administering ferroptosis inhibitor ferrostatin‐1 or an iron chelator can mitigate iron overload in the spinal cord of rats with sciatic nerve chronic compression injury, thereby preventing pain hypersensitivity associated with ferroptosis [9, 21]. This suggests a strong link between NP and increased intracellular Fe^2+^ levels due to disrupted iron metabolism during ferroptosis.

This investigation revealed that SNL model rats developed pronounced mechanical allodynia and thermal hyperalgesia, concomitant with a substantial elevation of Fe^2+^ concentrations within the spinal cord tissue. Furthermore, the administration of ferrostatin‐1 via intraperitoneal injection markedly attenuated these pain‐related hypersensitivities and also reduced Fe^2+^ deposition in the spinal cords of SNL rats. A comparable therapeutic efficacy was observed following Tuina intervention. In the pathogenesis of NP, in addition to morphological changes in neurons [34], ferroptosis‐induced neuronal injury displayed typical morphological characteristics, including mitochondrial atrophy, increased membrane density, and reduced or disappeared cristae [35]. Significant structural alterations were observed in the dorsal horn neurons of the spinal cord in SNL rats, including nuclear condensation, degeneration, disordered tissue structure, mitochondrial swelling, reduced cristae, partial dissolution or disappearance, membrane rupture, and increased density, consistent with previous findings [12]. Furthermore, a significant improvement in the structural integrity of dorsal horn neurons and their mitochondria was observed following treatment with either ferrostatin‐1 or Tuina, suggesting that Tuina may contribute to the inhibition of ferroptosis and the facilitation of neuronal repair.

The spinal dorsal horn was selected as the primary region of interest in this study because it represents the first central synaptic relay station for nociceptive afferent input, where primary afferent terminals establish synaptic contacts with second‐order neurons to initiate central pain processing [22, 36]. After peripheral nerve injury, this region undergoes prominent pathological remodeling, including neuronal hyperexcitability [37, 38], glial activation [39, 40], and neuroinflammatory responses [41, 42], which collectively contribute to the development and maintenance of central sensitization during NP. Therefore, examining molecular and structural alterations in the spinal dorsal horn provides important insights into the central mechanisms underlying NP.

Iron‐dependent lipid peroxidation represents another direct mechanism, alongside iron overload, that initiates ferroptosis and contributes to pain progression [8, 43]. Functioning as a pivotal proferroptotic protein, ACSL4 primarily facilitates the integration of polyunsaturated fatty acids into phospholipids, a crucial step for cell membrane synthesis [44]. Another significant component is GPX4, an essential antioxidant enzyme that participates in defensive responses against oxidation and modulates the ferroptosis cascade [45]. Therapeutically, enhancing GPX4 expression to inhibit ferroptosis reduces the reactivity of spinal cord dorsal horn neurons and astrocytes, thereby alleviating pain hypersensitivity in a chronic sciatic nerve compression injury model [21]. Recent evidence suggests that intracellular iron overload promotes the depletion of GPX4 and the increase of ACSL4, disrupting the balance between antioxidant and oxidative actions, thereby exacerbating lipid peroxidation‐induced oxidative damage in nerve cells [45]. Therefore, the upregulation of ACSL4 and MDA and the downregulation of GPX4 reflect the degree of lipid peroxidation and its involvement in pain processes [46, 47]. Consistently, this study confirmed these findings and further demonstrated that an association between Tuina intervention and a reduction in the expression of ACSL4 and MDA alongside an upregulation of GPX4, suggesting its potential involvement in antioxidant and neuroprotective processes.

The NCOA4‐FTH1‐LC3 pathway is recognized as a principal regulator of ferritinophagy. This autophagic degradation of ferritin is a fundamental driver of elevating intracellular Fe^2+^ and ROS, consequently driving the onset of ferroptosis [48]. The upregulation of NCOA4‐mediated ferritinophagy leads to a decrease in FTH1 levels and an increase in LC3‐II/LC3‐I and Fe^2+^ levels. Specific knockdown of NCOA4 significantly inhibits ferritinophagy [49, 50]. Therefore, blocking the NCOA4‐FTH1‐LC3 pathway‐mediated ferritinophagy is an effective strategy to suppress ferroptosis [51]. In line with previous results, this experimental silencing of NCOA4 in SNL rats led to a substantial reduction in spinal cord levels of NCOA4, the LC3‐II/LC3‐I ratio, and Fe^2+^, while concurrently upregulating FTH1 expression. This intervention suppressed the co‐expression of NCOA4 and FTH1, which correlated with a significant amelioration of pain behaviors. This study also found that Tuina intervention was associated with alterations in these key components of the ferritinophagy pathway. It is important to note that the observed correlation between Tuina’s effects and the modulation of the NCOA4‐FTH1‐LC3 pathway suggests a possible link but does not establish direct causation. The findings of this study mainly indicate that an association between Tuina treatment and changes in this pathway, which is known to be involved in ferroptosis. Direct causal evidence linking Tuina to the specific inhibition of NCOA4‐mediated ferroautophagy requires further investigation.

In the present study, the mechanistic investigation was primarily focused on the spinal level. The spinal dorsal horn serves as a critical integration center for peripheral nociceptive signals and plays a key role in the generation of central sensitization following nerve injury. Therefore, examining ferroptosis‐related molecular events in this region allows direct evaluation of local pathological processes associated with NP. Although supraspinal structures such as the thalamus, periaqueductal gray, and cortex are also involved in pain perception and descending modulation, the investigation of these higher central nervous system regions would require additional experimental designs and was beyond the scope of the present study. Future studies may further explore whether Tuina exerts modulatory effects on these supraspinal pain regulatory circuits. It should be noted that although the SNL model was established unilaterally (left L5), Tuina and sham manipulations were applied bilaterally to the hindlimbs. This design was adopted to better reflect clinical Tuina practice, in which bilateral stimulation of lower‐limb acupoints along the Gallbladder Meridian is commonly used in the management of NP [52]. In addition, bilateral manipulation helped maintain consistent handling conditions across animals. Previous studies have shown that contralateral electroacupuncture can significantly attenuate NP and, in some cases, produce stronger analgesic effects than ipsilateral stimulation [53], suggesting that acupuncture‐related analgesia may involve bilateral or central neuromodulatory mechanisms rather than purely local effects. Therefore, although bilateral manipulation was used primarily to mimic clinical practice and standardize experimental handling, it is possible that such stimulation may also induce contralateral or systemic modulation through spinal or supraspinal pain regulatory circuits. Consequently, while the present findings support the involvement of spinal ferroptosis–related mechanisms, the potential contribution of broader neuroregulatory pathways cannot be completely excluded.

Although these experimental results support the association between Tuina (and NCOA4 silencing) and the suppression of ferroptosis, this proposed mechanistic link requires further validation. A key limitation is that the evidence remains primarily associative; direct interventional proof linking Tuina to the specific inhibition of NCOA4‐mediated ferritinophagy is lacking. Moreover, the exclusive reliance on a rat model constrains translational relevance, and the long‐term sustainability of therapeutic effects remains unexplored. Finally, the precise upstream/downstream signaling events are not fully elucidated. A more detailed exploration of the underlying pathways and validation in other models are therefore warranted to substantiate the conclusions.

## 5. Conclusions

The analgesic effect of Tuina on SNL rats was associated with the inhibition of ferroptosis markers in the spinal cord dorsal horn. This effect may be linked to the modulation of iron metabolism and lipid peroxidation pathways, potentially involving the NCOA4‐mediated ferritinophagy axis. These results highlight a potential mechanism and provide a foundation for future research, but their direct clinical applicability requires further investigation.

## Author Contributions

Kailong Wang and Peipei Yang: conceptualization; data curation; formal analysis; methodology; investigation; drafted the paper; and writing–review and editing.

Xiaofeng Gan: data curation and investigation.

Hongliang Tang: methodology and supervision.

Liangyuan Tan: conceptualization; methodology; and writing–review and editing.

Yueqiang Hu: conceptualization and design of the study.

Kailong Wang and Peipei Yang contributed equally to this work.

## Funding

This study was supported by the National Natural Science Foundation of China (Grant Nos. 82060902, 82260961, and 82505721); the Construction Project of the Seventh Batch of Guangxi Clinical Medical Research Center‐“Guangxi Clinical Medical Research Center for Traditional Chinese Medicine Rehabilitation” (No. Guike Fa [2025] 293); and the Natural Science Foundation of Guangxi University of Chinese Medicine (Grant No. 2025QN023).

## Disclosure

All authors have read and approved the final manuscript.

## Conflicts of Interest

The authors declare no conflicts of interest.

## Data Availability

The datasets used and/or analyzed during the current study are available from the corresponding author on reasonable request.

## References

[1] Raja S. N. , Carr D. B. , Cohen M. et al., The Revised International Association for the Study of Pain Definition of Pain: Concepts, Challenges, and Compromises, Pain. (2020) 161, no. 9, 1976–1982, 10.1097/j.pain.0000000000001939. 32694387 PMC7680716

[2] Attal N. , Bouhassira D. , and Colvin L. , Advances and Challenges in Neuropathic Pain: a Narrative Review and Future Directions, British Journal of Anaesthesia. (2023) 131, no. 1, 79–92, 10.1016/j.bja.2023.04.021. 37210279

[3] Thouaye M. and Yalcin I. , Neuropathic Pain: from Actual Pharmacological Treatments to New Therapeutic Horizons, Pharmacology & Therapeutics. (2023) 251, 10.1016/j.pharmthera.2023.108546. 37832728

[4] Spagna A. and Attal N. , Pharmacotherapy and Noninvasive Neurostimulation for Neuropathic Pain, Presse Medicale. (2024) 53, no. 2, 10.1016/j.lpm.2024.104233. 38636787

[5] Zhu X. , Li F. , Wang M. et al., Integrated Analysis of Omics Data Reveal AP-1 as a Potential Regulation Hub in the Inflammation-Induced Hyperalgesia Rat Model, Frontiers in Immunology. (2021) 12, 10.3389/fimmu.2021.672498. PMC8194263 34122430

[6] Li L. , Guo L. L. , Gao R. et al., Ferroptosis: a New Regulatory Mechanism in Neuropathic Pain, Frontiers in Aging Neuroscience. (2023) 15, 10.3389/fnagi.2023.1206851. PMC10556472 37810619

[7] Kumari A. and Chauhan A. K. , Iron Nanoparticles as a Promising Compound for Food Fortification in Iron Deficiency Anemia: a Review, Journal of Food Science and Technology. (2022) 59, no. 9, 3319–3335, 10.1007/s13197-021-05184-4. 34219805 PMC8234770

[8] Liu S. , Gao X. , and Zhou S. , New Target for Prevention and Treatment of Neuroinflammation: Microglia Iron Accumulation and Ferroptosis, ASN Neuro. (2022) 14, no. 1, 10.1177/17590914221133236. PMC9607999 36285433

[9] Xu W. , Liu W. , and Yu W. , The Involvement of Iron Responsive Element (-) Divalent Metal Transporter 1-mediated the Spinal Iron Overload via CXCL10/CXCR3 Pathway in Neuropathic Pain in Rats, Neuroscience Letters. (2019) 694, 154–160, 10.1016/j.neulet.2018.12.001, 2-s2.0-85057757105. 30521948

[10] Din M. A. U. , Lin Y. , Wang N. , Wang B. , and Mao F. , Ferroptosis and the ubiquitin-proteasome System: Exploring Treatment Targets in Cancer, Frontiers in Pharmacology. (2024) 15, 10.3389/fphar.2024.1383203. PMC11043542 38666028

[11] Hoelzgen F. , Nguyen T. T. P. , Klukin E. et al., Structural Basis for the Intracellular Regulation of Ferritin Degradation, Nature Communications. (2024) 15, no. 1, 10.1038/s41467-024-48151-1. PMC11076521 38714719

[12] Ryan F. , Blex C. , Ngo T. D. et al., Ferroptosis Inhibitor Improves Outcome After Early and Delayed Treatment in Mild Spinal Cord Injury, Acta Neuropathologica. (2024) 147, no. 1, 10.1007/s00401-024-02758-2. PMC11193702 38907771

[13] Wang H. , Liu X. , Jing X. et al., Oxidative Stress Activates YAP/TEAD1/NCOA4 Axis to Promote Ferroptosis of Endplate Chondrocytes and Aggravate Intervertebral Disc Degeneration, Journal of Orthopaedic Translation. (2025) 54, 8–25, 10.1016/j.jot.2025.07.001. 40688343 PMC12274860

[14] Wan K. , Jia M. , Zhang H. et al., Electroacupuncture Alleviates Neuropathic Pain by Suppressing Ferroptosis in Dorsal Root Ganglion via SAT1/ALOX15 Signaling, Molecular Neurobiology. (2023) 60, no. 10, 6121–6132, 10.1007/s12035-023-03463-z. 37421564

[15] Tang B. , Xu X. , Zhao W. , Zhou Q. , Du G. , and Wang H. , Electroacupuncture Regulates Neuronal Ferroptosis and Ferritinophagy Through lysosomal-mediated TFEB Activation in Cerebral ischemia-reperfusion, Journal of Cerebral Blood Flow and Metabolism. (2025) . 10.1177/0271678X251399016 PMC12640282 41272418

[16] Xu H. , Wang Z. , Wang Z. et al., Recent Trends in Tuina for Chronic Pain Management: a Bibliometric Analysis and Literature Review, Complementary Therapies in Medicine. (2024) 84, 10.1016/j.ctim.2024.103068. 39004289

[17] Liu Z. , Wang H. , Yu T. et al., A Review on the Mechanism of Tuina Promoting the Recovery of Peripheral Nerve Injury, Evidence-Based Complementary and Alternative Medicine. (2021) 2021, 1–8, 10.1155/2021/6652099. PMC8275372 34285705

[18] Liu Z. F. , Wang H. R. , Yu T. Y. , Zhang Y. Q. , Jiao Y. , and Wang X. Y. , Tuina for peripherally-induced Neuropathic Pain: a Review of Analgesic Mechanism, Frontiers in Neuroscience. (2022) 16, 10.3389/fnins.2022.1096734. PMC9817144 36620462

[19] Wang Q. , Lin J. , Yang P. et al., Effect of Massage on the TLR4 Signalling Pathway in Rats with Neuropathic Pain, Pain Research and Management. (2020) 2020, 1–6, 10.1155/2020/8309745. PMC7759416 33381249

[20] Shu H. W. , Clinical Observation on Acupuncture Treatment of Piriformis syndrome, Journal of Traditional Chinese Medicine. (2003) 23. 12747195

[21] Wang H. , Huo X. , Han C. et al., Ferroptosis is Involved in the Development of Neuropathic Pain and Allodynia, Molecular and Cellular Biochemistry. (2021) 476, no. 8, 3149–3161, 10.1007/s11010-021-04138-w. 33864570

[22] Antal M. , Molecular Anatomy of Synaptic and Extrasynaptic Neurotransmission Between Nociceptive Primary Afferents and Spinal Dorsal Horn Neurons, International Journal of Molecular Sciences. (2025) 26, no. 5, 10.3390/ijms26052356. PMC11900602 40076973

[23] Gomez K. , Vargas-Parada A. , Duran P. et al., L5-6 Spinal Nerve Ligation-induced Neuropathy Changes the Location and Function of Ca(2+) Channels and Cdk5 and Affects the Compound Action Potential in Adjacent Intact L4 Afferent Fibers, Neuroscience. (2021) 471, 20–31, 10.1016/j.neuroscience.2021.07.013. 34303780 PMC8384716

[24] Tong S. H. , Zhou J. , Ye F. et al., Activation of TLR4/CCL2 in Intact Neurons Drives Radicular Injury-Induced Global Nerve Trunk Hypersensitivity in Radiculopathy Preclinical Models, Journal of Pain Research. (2025) 18, 3903–3918, 10.2147/jpr.s499997. 40786564 PMC12333639

[25] Shibata Y. , Matsumoto Y. , Kohno K. , Nakashima Y. , and Tsuda M. , Microgliosis in the Spinal Dorsal Horn Early After Peripheral Nerve Injury Is Associated with Damage to Primary Afferent Aβ-Fibers, Cells. (2025) 14, no. 9, 10.3390/cells14090666. PMC12071663 40358190

[26] Wang X. , Gao Y. , Qiao Y. , Yv L. , Li L. , and Xu J. T. , Peripheral Nerve injury-induced Upregulation of FKBP5 in the Spinal Dorsal Horn via Activating NF-κB Pathway Aggravates Neuropathic Pain in Rats, International Immunopharmacology. (2025) 162, 10.1016/j.intimp.2025.115124. 40544667

[27] Xue T. , Song Y. , Zhao J. , Fan G. , and Liu Z. , Inhibition of S100A4 Decreases Neurotoxic Astrocyte Reactivity and Attenuates Neuropathic Pain via the TLR4/NF-κB Pathway in a Rat Model of Spinal Nerve Ligation, The Journal of Headache and Pain. (2025) 26, no. 1, 10.1186/s10194-025-02045-9. PMC12044810 40312684

[28] Livak K. J. and Schmittgen T. D. , Analysis of Relative Gene Expression Data Using real-time Quantitative PCR and the 2(-Delta Delta C(T)) Method, Methods. (2001) 25, no. 4, 402–408, 10.1006/meth.2001.1262, 2-s2.0-0035710746. 11846609

[29] Shan Y. and Mollereau B. , Non-Canonical Functions of Regulated Cell Death Machinery Regulate Cellular Growth, Invasion and the Interplay between Cell Death Modalities, Frontiers in Cell Death. (2024) 3, 10.3389/fceld.2024.1423805.

[30] Bu Z. Q. , Yu H. Y. , Wang J. et al., Emerging Role of Ferroptosis in the Pathogenesis of Ischemic Stroke: a New Therapeutic Target?, ASN Neuro. (2021) 13, no. 1, 10.1177/17590914211037505. PMC8424725 34463559

[31] Van Kessel K. and Cosa G. , Lipid-Derived Electrophiles Inhibit the Function of Membrane Channels During Ferroptosis, Proceedings of the National Academy of Sciences of the United States of America. (2024) 121, no. 21. 10.1073/pnas.2317616121 PMC11127018 38743627

[32] Wang Y. , Li H. , He Q. , Zou R. , Cai J. , and Zhang L. , Ferroptosis: Underlying Mechanisms and Involvement in Neurodegenerative Diseases, Apoptosis. (2024) 29, no. 1-2, 3–21, 10.1007/s10495-023-01902-9. 37848673

[33] Liao C. , Guo J. , Li S. et al., Ferroptosis Regulated by 5-HT3a Receptor via Calcium/Calmodulin Signaling Contributes to Neuropathic Pain in Brachial Plexus Avulsion Rat Models, ACS Chemical Neuroscience. (2024) . 10.1021/acschemneuro.4c00499 39370752

[34] Xue C. , Kui W. , Huang A. et al., Electroacupuncture Suppresses Neuronal Ferroptosis to Relieve Chronic Neuropathic Pain, Journal of Cellular and Molecular Medicine. (2024) 28, no. 7, 10.1111/jcmm.18240. PMC10955159 38509741

[35] Tang L. , Liu S. , Li S. , Chen Y. , Xie B. , and Zhou J. , Induction Mechanism of Ferroptosis, Necroptosis, and Pyroptosis: a Novel Therapeutic Target in Nervous System Diseases, International Journal of Molecular Sciences. (2023) 24, no. 12, 10.3390/ijms241210127. PMC10299044 37373274

[36] Zhang Z. , Zheng H. , Yu Q. , and Jing X. , Understanding of Spinal Wide Dynamic Range Neurons and Their Modulation on Pathological Pain, Journal of Pain Research. (2024) 17, 441–457, 10.2147/jpr.s446803. 38318328 PMC10840524

[37] Huang Y. , Chen S. R. , Chen H. , Zhou J. J. , Jin D. , and Pan H. L. , Theta-Burst Stimulation of Primary Afferents Drives Long-Term Potentiation in the Spinal Cord and Persistent Pain via alpha2delta-1-Bound NMDA Receptors, Journal of Neuroscience. (2021) 42, no. 3, 513–527, 10.1523/jneurosci.1968-21.2021. 34880118 PMC8802928

[38] Koga K. , Kobayashi K. , and Tsuda M. , Voltage-Gated Calcium Channel Subunit alpha2delta-1 in Spinal Dorsal Horn Neurons Contributes to Aberrant Excitatory Synaptic Transmission and Mechanical Hypersensitivity After Peripheral Nerve Injury, Frontiers in Molecular Neuroscience. (2023) 16. 10.3389/fnmol.2023.1099925 PMC10076860 37033377

[39] Kohno K. and Tsuda M. , Neuron-Microglia Interactions Modulating Neuropathic Pain, International Immunology. (2025) 37, no. 10, 589–598, 10.1093/intimm/dxaf022. 40251994

[40] Shan L. , Xu K. , Ji L. , Zeng Q. , Liu Y. , Wu Y. , Chen Y. , Li Y. , Hu Q. , Wu J. , Xu Y. , Luo Y. , Li C. , Wu C. , Jiang C. , and Wang Z. , Injured Sensory neurons-derived galectin-3 Contributes to Neuropathic Pain via Programming Microglia in the Spinal Dorsal Horn, Brain, Behavior, and Immunity. (2024) 117, 80–99, 10.1016/j.bbi.2024.01.002. 38190982

[41] Mu Y. , Mei Y. , Chen Y. et al., Perisciatic Nerve Dexmedetomidine Alleviates Spinal Oxidative Stress and Improves Peripheral Mitochondrial Dynamic Equilibrium in a Neuropathic Pain Mouse Model in an AMPK-Dependent Manner, Disease Markers. (2022) 2022, 1–14, 10.1155/2022/6889676. PMC9236761 35769812

[42] Stojanovic B. S. , Bevc I. M. , Stojanovic M. D. et al., Oxidative Stress, Inflammation, and Cellular Senescence in Neuropathic Pain: Mechanistic Crosstalk, Antioxidants. (2025) 14, no. 10, 10.3390/antiox14101166. PMC12561125 41154475

[43] Liu T. , Wang R. , Qi W. et al., Methyl Ferulic Acid Alleviates Neuropathic Pain by Inhibiting Nox4-induced Ferroptosis in Dorsal Root Ganglia Neurons in Rats, Molecular Neurobiology. (2023) 60, no. 6, 3175–3189, 10.1007/s12035-023-03270-6. 36813954

[44] Cheng H. , Wang M. , Su J. et al., Lipid Metabolism and Cancer, Life (Basel). (2022) 12, no. 6, 10.3390/life12060784. PMC9224822 35743814

[45] Fan S. , Wang K. , Zhang T. et al., Mechanisms and Therapeutic Potential of GPX4 in Pain Modulation, Pain and Therapy. (2025) 14, no. 1, 21–45, 10.1007/s40122-024-00673-8. 39503961 PMC11751247

[46] An S. S. , Kim Y. O. , Park C. H. , Lin H. , and Yoon M. H. , Antiallodynic Effect of Intrathecal epigallocatechin-3-gallate due to Suppression of Reactive Oxygen Species, Korean Journal of Anesthesiology. (2014) 67, no. 2, 123–128, 10.4097/kjae.2014.67.2.123, 2-s2.0-84907149572. 25237449 PMC4166384

[47] Guo Y. , Du J. , Xiao C. et al., Inhibition of Ferroptosis-like Cell Death Attenuates Neuropathic Pain Reactions Induced by Peripheral Nerve Injury in Rats, European Journal of Pain. (2021) 25, no. 6, 1227–1240, 10.1002/ejp.1737. 33497529

[48] Liu X. , Xie C. , Wang Y. et al., Ferritinophagy and Ferroptosis in Cerebral Ischemia Reperfusion Injury, Neurochemical Research. (2024) 49, no. 8, 1965–1979, 10.1007/s11064-024-04161-5. 38834843 PMC11233298

[49] Yuan Z. , Zhou X. , Zou Y. et al., Hypoxia Aggravates Neuron Ferroptosis in Early Brain Injury Following Subarachnoid Hemorrhage via NCOA4-Meditated Ferritinophagy, Antioxidants. (2023) 12, 10.3390/antiox12122097. PMC10740655 38136217

[50] Xiu Z. , Zhu Y. , Han J. et al., Caryophyllene Oxide Induces Ferritinophagy by Regulating the NCOA4/FTH1/LC3 Pathway in Hepatocellular Carcinoma, Frontiers in Pharmacology. (2022) 13, 10.3389/fphar.2022.930958. PMC9313605 35899120

[51] Hou W. , Xie Y. , Song X. et al., Autophagy Promotes Ferroptosis by Degradation of Ferritin, Autophagy. (2016) 12, 1425–1428, 10.1080/15548627.2016.1187366, 2-s2.0-84976292806. 27245739 PMC4968231

[52] Fan Z. , Jia S. , Zhou X. et al., Clinical Efficacy of Tuina Therapy Combined with Traditional Chinese Exercises in the Treatment of Symptomatic Lumbar Disc Herniation: a Multicentre Randomised Controlled Trial Protocol, Frontiers in Neurology. (2025) 16, 10.3389/fneur.2025.1497933. PMC11802433 39926017

[53] Zhang H. , Sun J. , Xin X. , Huo Z. , and Li D. , Contralateral Electroacupuncture Relieves Chronic Neuropathic Pain in Rats with Spared Nerve Injury, Medical Science Monitor. (2018) 24, 2970–2974, 10.12659/msm.909741, 2-s2.0-85046680684. 29735969 PMC5963317

